# Assessing Liver Fibrosis in Type 2 Diabetes Mellitus Patients With Metabolic Dysfunction-Associated Steatotic Liver Disease: The Role of Non-invasive Scoring Systems and Associated Factors

**DOI:** 10.7759/cureus.62405

**Published:** 2024-06-14

**Authors:** Arindam Naskar, Agnibho Mondal, Rupak Chatterjee, Ruchika D De, Sasmit Roy

**Affiliations:** 1 Department of Endocrinology, Nutrition, and Metabolic Diseases, School of Tropical Medicine, Kolkata, IND; 2 Department of Infectious Diseases and Advanced Microbiology, School of Tropical Medicine, Kolkata, IND; 3 Department of Tropical Medicine, School of Tropical Medicine, Kolkata, IND; 4 Department of Nephrology, University of Virginia, Lynchburg, USA

**Keywords:** diabetes, metabolic syndrome, liver fibrosis, masld, t2dm

## Abstract

Background

Metabolic dysfunction-associated steatotic liver disease (MASLD) constitutes a significant cause of chronic liver disease globally. Type 2 diabetes mellitus (T2DM) is a crucial risk factor for MASLD. This investigation is aimed at assessing hepatic fibrosis in T2DM patients with MASLD.

Methods

This cross-sectional study focused on T2DM patients with MASLD attending a tertiary care center in eastern India. Exclusion criteria were chronic alcohol intake (more than 21 units/week for males and more than 14 units/week for females), other chronic liver diseases, and pregnancy. The study utilized abdominal ultrasonography and transient elastography, complemented by calculating the BARD score, nonalcoholic fatty liver disease (NAFLD) fibrosis score, aspartate aminotransferase to platelet ratio index (APRI) score, and fibrosis 4 (FIB-4) index. The prevalence of advanced fibrosis in patients with T2DM and MASLD was assessed using transient elastography.

Results

Among the 149 T2DM patients with MASLD studied, 59.7% were female, with an average age of 49.09 years and a T2DM duration of 7.3 years. Transaminitis was detected in 9.4% of the subjects. The risk assessment of hepatic fibrosis revealed that 14.1% of patients had a high risk of hepatic fibrosis on BARD scoring, the NAFLD fibrosis score was in the range of F3-F4 in 8.7% of patients, the FIB-4 index showed a high risk of fibrosis in 5.4% of patients, and the APRI scoring showed severe fibrosis in 3.4% of patients. The prevalence of advanced fibrosis in patients with T2DM and MASLD was 7.4% (95% confidence interval (CI) 3.7 to 12.8), while 75.8% (95% CI 68.2 to 82.5) of participants had at least some level of hepatic fibrosis as measured by transient elastography. Notably, there was a significant positive correlation between these scores and the duration of diabetes and serum bilirubin levels, as corroborated by concordant transient elastography findings.

On multivariate logistic regression, systolic blood pressure, serum total bilirubin level, and serum aspartate aminotransferase level had significant predictive value for advanced hepatic fibrosis.

Conclusion

The significant predictive value of systolic blood pressure, serum total bilirubin level, and serum aspartate aminotransferase level for hepatic fibrosis emphasizes the importance of integrated monitoring for these patients.

## Introduction

Diabetes mellitus, a chronic metabolic syndrome affecting millions worldwide, imposes a substantial burden on healthcare systems globally. Type 2 diabetes mellitus (T2DM), which is marked by hyperglycemia due to insulin resistance and insufficient insulin secretion, represents a significant percentage of diabetes cases. Diabetes mellitus is affecting around 537 million adults aged 20 to 79 years worldwide and is estimated to affect almost 643 million by 2030 and 783 million by 2045, according to the census by the International Diabetes Federation, thus proving to be a global public health burden to the healthcare system [1].

In recent years, metabolic dysfunction-associated steatotic liver disease (MASLD) has emerged as one of the most prevalent chronic liver diseases associated with T2DM, fostering a bidirectional relationship that underscores its burgeoning significance as a global health concern [2]. MASLD, a multifactorial condition influenced by factors such as an unhealthy lifestyle, obesity, dyslipidemia, and metabolic syndrome, encompasses a spectrum of liver disorders ranging from simple hepatic steatosis (nonalcoholic fatty liver (NAFL)) to non-alcoholic steatohepatitis (NASH), characterized by hepatocyte inflammation and ballooning, with potential progression to liver fibrosis, cirrhosis, and hepatocellular carcinoma [3].

The prevalence of MASLD among patients with T2DM is staggering, with reports indicating a prevalence of 55.5%. The prevalence of advanced fibrosis in T2DM patients with MASLD is estimated to be 17% [4].T2DM accelerates the development of advanced fibrosis in MASLD, while MASLD increases the risk of developing T2DM twofold, illustrating the complex interplay between these two conditions [5].

Despite advancements in diagnostic tools, including various fibrosis markers and imaging modalities, challenges persist in accurately assessing and managing MASLD in patients with T2DM. Furthermore, the recent designation of metabolic dysfunction-associated fatty liver disease (MAFLD) underscores the intertwined nature of fatty liver disease and metabolic disorders, necessitating a comprehensive approach to evaluation and management. Beyond its hepatic manifestations, MASLD is increasingly recognized as a multisystem disorder, predisposing individuals to a spectrum of extrahepatic complications, including cardiovascular disease and malignancies, further emphasizing its broader impact on health outcomes [6].

This article aims to explore the prevalence of advanced hepatic fibrosis in patients with T2DM and MASLD, while also investigating potential risk factors associated with the development of these complications. Through a rigorous examination of existing literature and empirical data, we endeavor to enhance our understanding of the intricate relationship between T2DM and MASLD, with implications for clinical practice and future research.

## Materials and methods

A cross-sectional study was conducted at the School of Tropical Medicine, Kolkata, India among patients with T2DM and MASLD attending the diabetic outpatient department of the institute. The objective of the study was to find the prevalence of hepatic fibrosis by transient elastography, to assess factors associated with hepatic fibrosis, and to evaluate the correlation between non-invasive markers of fibrosis and transient elastography findings in patients with T2DM with MASLD. Patients with a history of significant alcohol consumption (defined as more than 21 units/week for males and more than 14 units/week for females), chronic liver disease, and pregnant females were excluded from participation.

Each participant underwent comprehensive clinical and investigational assessments, with data recorded for variables including age, sex, height, weight, duration of diabetes, medication history (oral hypoglycaemic agents or insulin), hypertension, dyslipidemia, smoking or alcohol consumption, and other complications. Laboratory investigations encompassed complete blood counts, fasting blood glucose, HbA1c, lipid profile, liver function tests (LFTs), uric acid levels, and, where deemed necessary, prothrombin time/international normalized ratio (PT/INR). Ultrasonography of the abdomen was performed for all patients, and fibrosis extent was evaluated using transient elastography [7].

Body mass index (BMI) was computed using the formula: weight (in kilograms)/height (in meters squared). Additionally, the BARD score [8], nonalcoholic fatty liver disease (NAFLD) fibrosis score [8], fibrosis 4 (FIB-4) index [9], and aspartate aminotransferase to platelet ratio index (APRI) score [10] were calculated for each participant.

The BARD score assigns points based on BMI, aspartate aminotransferase/alanine aminotransferase (AST/ALT) ratio, and the presence of newly diagnosed or pre-existing T2DM. The NAFLD fibrosis score incorporates variables such as age, hyperglycemia, BMI, platelet count, albumin level, and AST/ALT ratio. Meanwhile, the FIB-4 index considers age, platelet count, ALT level, and AST level.

Transient elastography was conducted with a Fibrotouch FT100 with a dynamic probe. Taking the expected prevalence of advanced fibrosis as 17% [7] among the T2DM patients with MASLD, a minimum sample size of 129 was calculated. We exceeded this sample size to increase the power of the study. The study participants were recruited by convenience sampling.

Descriptive statistics, including mean, standard deviation, frequency, and percentages, were employed to summarize the data. Missing values were imputed using nonparametric missing value imputation via random forest methodology. Continuous variables were compared using the T-test, while categorical variables were assessed using Fisher's exact test. Spearman rank correlation was applied to test for associations between variables. The predictive values of different parameters were evaluated using logistic regression models.

A significance level of p<0.05 was adopted, and all statistical analyses were performed using R software version 4.3.2 developed by the R Foundation for statistical computing.

Ethical permission for this study was obtained from the Clinical Research Ethics Committee of the School of Tropical Medicine, Kolkata. Informed consent was obtained before the recruitment of study participants.

## Results

The study encompassed 149 patients, with a notable predominance of females, constituting 59.7% of the cohort. The average age of the population was 49.09 years, with a mean duration of type 2 diabetes mellitus (T2DM) of 7.3 years. Of the participants, 68.5% (n=102) were solely on oral hypoglycemic agents, while 31.5% (47 out of 149) were prescribed both insulin and oral hypoglycemics. The majority of the study participants, i.e., 88.6% (132 out of 149) were on statin therapy. Smoking was reported by 5.4% (n=8) of the patients, while no history of alcohol consumption was noted. The majority of patients (90.6%, n=142) exhibited liver enzyme levels within normal limits, with both aspartate aminotransferase (AST) and alanine transaminase (ALT) below 40 IU/L. The study parameters are summarized in Tables 1, 2.

**Table 1:** Summary of numerical parameters WBC: white blood cell; AST: aspartate aminotransferase; ALT: alanine aminotransferase; NAFLD: nonalcoholic fatty liver disease; FIB-4: fibrosis 4; APRI: aspartate aminotransferase to platelet ratio index

Parameter	Findings [Mean± SD]
Age (years)	49.09± 8.54
Body mass index (kg/m^2^)	26.32± 3.69
Duration of diabetes (years)	7.3± 5.46
Systolic blood pressure (mmHg)	135.2± 16.85
Diastolic blood pressure (mmHg)	82.1± 9.27
Fasting blood sugar (mg/dl)	163.8± 65
HbA1C (%)	8.02± 1.43
Hemoglobin (g/dl)	12.42± 1.42
WBC (cells/microliter)	9302± 2103.6
Platelet (cells/microliter)	204897± 62888
Total bilirubin (mg/dl)	0.68± 0.38
Direct bilirubin (mg/dl)	0.31± 0.16
Albumin (g/dl)	4.3± 0.35
Globulin (g/dl)	3.26± 0.45
AST (IU/L)	25.43± 14.38
ALT (IU/L)	29.22± 24.42
Alkaline phosphatase (IU/L)	114.1± 39.3
Serum cholesterol (mg/dl)	170.7± 42.6
Triglyceride (mg/dl)	214.1± 118
High density lipoprotein (mg/dl)	44.58 ± 7.25
Low density lipoprotein (mg/dl)	96.92± 28.8
Transient elastography measurements (kPa)	7.1± 2.75
BARD score	1.93± 0.59
NAFLD fibrosis score	-0.81± 1.1
FIB-4 index	1.3± 0.8
APRI score	0.4±0.2

**Table 2:** Summary of categorical parameters OHA: oral hypoglycemic agent; NAFLD: nonalcoholic fatty liver disease; FIB-4: fibrosis 4; APRI: aspartate aminotransferase to platelet ratio index

Parameter		Count	Percentage
Gender	Female	89	59.7
	Male	60	40.3
Diabetes management	Both insulin and OHA	47	31.5
	OHA Only	102	68.5
Dyslipidaemia		94	63.1
Smoker		8	5.4
BARD score	Low risk (Score 0 -1)	128	85.9
	High risk (Score 2 – 4)	21	14.1
NAFLD fibrosis score	F0 – F2 (Score)	34	22.8
	Indeterminate score (Score -1.455 to 0.675)	102	68.5
	F3 – F4 (Score >0.675)	13	8.7
FIB-4 index	Low risk of fibrosis (<1.3)	96	64.4
	Indeterminate risk of fibrosis (1.3-2.67)	45	30.2
	High risk of fibrosis (≥2.67)	8	5.4
APRI score	No significant fibrosis	133	89.3
	Significant fibrosis	11	7.4
	Severe fibrosis	5	3.4
Transient elastography	No fibrosis (F0, < 5.5)	36	24.2
	Mild fibrosis (F1, 5.5-8)	77	51.7
	Moderate fibrosis (F2, < 8-10)	25	16.8
	Severe fibrosis (F3, 11-16)	8	5.4
	Cirrhosis (F4, >16)	3	2

Among the study participants, 21 (14.1%) patients had an elevated risk of hepatic fibrosis on BARD scoring. The NAFLD fibrosis score was in the range of F3-F4 in 13 (8.7%) patients. The FIB-4 index showed a high risk of fibrosis in eight (5.4%) patients. APRI scoring of the study participants showed that severe fibrosis was present in five (3.4%) patients, while significant fibrosis was present in 11 (7.4%) patients.

The prevalence of advanced hepatic fibrosis in the study population was found to be 7.4% (95% confidence interval (CI) 3.7 to 12.8) using transient elastography ( ≥11 kPa). Our findings showed that 75.8% (95% CI 68.3 to 82) participants had at least some level of fibrosis, with mild fibrosis in 51.7% (95% CI 43.4 to 59.9) and moderate fibrosis in 16.8% (95% CI 11.2 to 23.8) participants.

Correlational analyses revealed significant associations between the higher NAFLD fibrosis score and the duration of diabetes (p<0.001), total bilirubin (p<0.001), direct bilirubin (p<0.001), and transient elastography measurements (p=0.03) while it was negatively correlated with WBC count (p=0.02). Notably, the NAFLD fibrosis score exhibited no correlation with control of blood sugar.

Similarly, a higher FIB-4 score correlated significantly with the duration of diabetes (p=0.002), total bilirubin (p<0.001), direct bilirubin (p<0.001), and transient elastography measurements (p<0.001) while it was negatively correlated with WBC count (p=0.01). Conversely, there was no correlation between the FIB-4 score and control of blood sugar.

The APRI score demonstrated significant positive correlations with total bilirubin (p=0.002) and conjugated bilirubin (p=0.001) while it was negatively correlated with WBC count (p=0.002). Moreover, a positive correlation between the APRI score and transient elastography measurements was evident (p<0.001).

Strong correlations were observed between the NAFLD fibrosis score and both FIB-4 index (rho=0.773, p<0.001) and APRI score (rho=0.494, p<0.001), while the FIB-4 index and APRI score also exhibited a robust correlation (rho=0.829, p<0.001).

**Figure 1:**
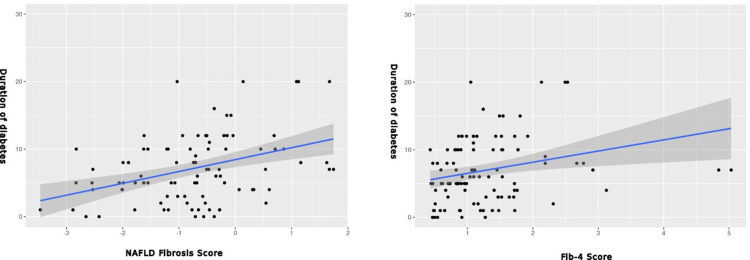
Correlation between duration of diabetes and fibrosis scoring

**Figure 2:**
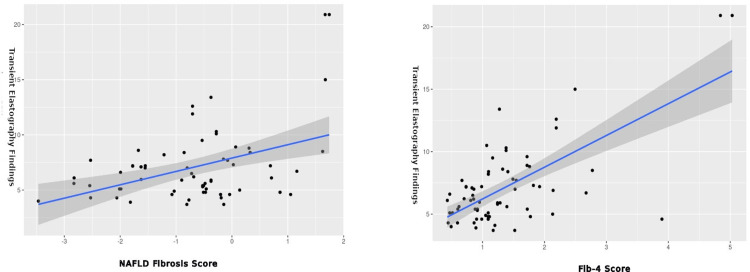
Correlation between transient elastography findings and fibrosis scoring

The correlation between fibrosis scoring and duration of diabetes is shown in Figure 1, while the correlation between transient elastography and non-invasive markers is shown in Figure 2.

Multivariate logistic regression identified elevated systolic blood pressure (p=0.003), elevated total bilirubin level (p=0.043), and elevated aspartate aminotransferase level (p=0.023) as significant predictors of advanced hepatic fibrosis as estimated by transient elastography. The logistic regression model is shown in Table 3.

**Table 3:** Multivariate logistic regression showing predictive parameters of advanced hepatic fibrosis on transient elastography

	Estimate	Standard error	Z-value	P-value
Intercept	-14.706	3.668	-4.01	<0.001
Systolic blood pressure	0.067	0.022	2.996	0.003
Total bilirubin	1.711	0.845	2.024	0.043
Aspartate aminotransferase	0.041	0.018	2.273	0.023

These findings shed light on the intricate relationships between various clinical and laboratory parameters in patients with T2DM and MASLD, underscoring the multifactorial nature of hepatic fibrosis progression in this population.

## Discussion

Patients with MASLD often present with nonspecific symptoms, with fatigue being a common complaint. Mild to moderate hepatomegaly represents the most prevalent clinical sign, while advanced stages of the disease spectrum may manifest signs of end-stage liver disease [11]. Although elevated serum aminotransferases may be observed in MASLD, liver function tests (LFTs) may appear normal in some cases, a phenomenon corroborated by our study [11]. While abdominal ultrasonography is commonly utilized to assess fatty liver, liver biopsy remains the gold standard for diagnosis. However, non-invasive markers such as the NAFLD fibrosis score (NFS), FIB-4 index, and other indices serve as valuable alternatives when liver biopsy is not feasible, as employed in our investigation [11].

The FIB-4 and NAFLD fibrosis scores exhibited a positive correlation with each other and with transient elastography findings, as anticipated. Total bilirubin was significantly correlated with the NAFLD fibrosis score, FIB-4 index, and APRI score, while the duration of diabetes showed a significant correlation with the NAFLD fibrosis score and FIB-4 index.

Among the study participants, advanced fibrosis was present in 7.4%, while 75.8% of patients had at least some level of fibrosis as determined by transient elastography.

We found that systolic blood pressure, elevated serum total bilirubin level, and elevated serum aspartate aminotransferase level had significant predictive value for hepatic fibrosis.

Although dyslipidemia may be associated with MASLD [12], we did not find a positive correlation between a deranged lipid profile and hepatic fibrosis scores. However, the majority of the study participants were on statin therapy, which may have a confounding effect.

We employed multiple non-invasive markers of hepatic fibrosis that measured different aspects of the condition, including the BARD score [8] to determine the risk of hepatic fibrosis, the NAFLD fibrosis score [8] to determine the grade of hepatic fibrosis, the FIB-4 index [9] to estimate the risk of fibrosis, and the APRI score [10] to assess the grade of fibrosis. As the markers measure distinct aspects of hepatic fibrosis in MASLD, there were variations in their interpretation. However, their correlation with each other indicates an underlying common pathological condition.

Although some prior studies [13,14] have noticed a positive correlation between WBC count and MASLD, our study showed that WBC count was negatively correlated with hepatic fibrosis patients with MASLD. The NAFLD fibrosis score, FIB-4 index, and APRI score showed a negative correlation with WBC count, although they had no correlation with transient elastography measurements. Further studies may be needed to find the reason for this varied finding.

A systematic review and meta-analysis underscored the substantial prevalence of advanced fibrosis in MASLD patients with type 2 diabetes mellitus (T2DM), with advanced fibrosis (F3-F4) observed in 15% of cases. However, our study reported a lower prevalence of advanced fibrosis (7.4%), suggesting potential variations across populations [4].

A similar study from India in 2023 showed a prevalence of advanced fibrosis in patients with T2DM and MASLD of 17% [7]. However, in our study, only 7.4% of patients were found to have advanced fibrosis by transient elastography findings. On the other hand, their study showed the presence of moderate fibrosis in 14.5% of cases, which was in agreement with the 16.8% prevalence of moderate fibrosis in our study participants.

Education regarding diet and lifestyle modifications is pivotal in the context of patient management, emphasizing food choices, proportions, and regular exercise during healthcare visits.

The significance of our study lies in its contribution to understanding the complex interplay between T2DM and MASLD, particularly concerning the development of hepatic fibrosis. By investigating factors associated with hepatic fibrosis in patients with T2DM and MASLD, our study sheds light on potential predictors, including systolic blood pressure and biochemical markers such as bilirubin and aspartate aminotransferase. This knowledge is crucial for clinicians to identify individuals at higher risk of developing advanced liver disease and tailor management strategies accordingly.

Moreover, our study adds to the existing literature by utilizing non-invasive tools such as transient elastography and various scoring systems to assess hepatic fibrosis in this population. This approach not only provides valuable insights into the prevalence and severity of fibrosis but also offers a practical alternative to liver biopsy, especially in settings where invasive procedures may not be feasible or desirable.

While our study is valuable in evaluating factors related to hepatic fibrosis development in T2DM patients with MASLD, limitations include the absence of a liver biopsy as a gold standard test, impacting the accuracy of scores. Nonetheless, our findings provide meaningful insights into managing these patients.

## Conclusions

In conclusion, the development of hepatic fibrosis in patients with T2DM and MASLD is influenced by numerous factors. Our study highlights that systolic blood pressure, total bilirubin, and aspartate aminotransferase hold significant predictive value for advanced hepatic fibrosis. These findings underscore the importance of recognizing the potential for hepatic fibrosis and understanding the associated factors to deliver effective medical care to diabetic patients. By acknowledging these factors, healthcare providers can optimize management strategies and enhance patient outcomes in this high-risk population.
